# Understanding How Nutrition Literacy Links to Dietary Adherence in Patients Undergoing Maintenance Hemodialysis: A Theoretical Exploration using Partial Least Squares Structural Equation Modeling

**DOI:** 10.3390/ijerph17207479

**Published:** 2020-10-14

**Authors:** Jun-Hao Lim, Karuthan Chinna, Pramod Khosla, Tilakavati Karupaiah, Zulfitri Azuan Mat Daud

**Affiliations:** 1 Department of Dietetics, Faculty of Medicine and Health Sciences, Universiti Putra Malaysia, UPM Serdang 43400, Malaysia; sinhao0624@yahoo.com; 2 School of Medicine, Faculty of Health and Medical Sciences, Taylor’s University, Subang Jaya 47500, Malaysia; Karuthan.Chinna@taylors.edu.my; 3 Department of Nutrition and Food Science, Wayne State University, Detroit, MI 48202-0340, USA; aa0987@wayne.edu; 4 School of Biosciences, Faculty of Health and Medical Sciences, Taylor’s University, Subang Jaya 47500, Malaysia; Tilakavati.Karupaiah@taylors.edu.my; 5 Research Center of Excellent (RCoE) Nutrition and Non-communicable Diseases, Faculty of Medicine and Health Sciences, Universiti Putra Malaysia, Serdang 43400, Malaysia

**Keywords:** hemodialysis, nutrition literacy, health literacy, dietary adherence, dietary knowledge, health belief, self-efficacy, self-management skills

## Abstract

Dietary non-adherence is pervasive in the hemodialysis (HD) population. Health literacy is a plausible predictor of dietary adherence in HD patients, but its putative mechanism is scarcely studied. Thus, this study aimed to establish the causal model linking nutrition literacy to dietary adherence in the HD population. This was a multi-centre, cross-sectional study, involving 218 randomly selected multi-ethnic HD patients from nine dialysis centres in Klang Valley, Malaysia. Dietary adherence and self-management skills were assessed using validated End-Stage Renal Disease Adherence Questionnaire and Perceived Kidney/Dialysis Self-Management Scale, respectively. Validated self-developed scales were used to gauge nutrition literacy, dietary knowledge and Health Belief Model constructs. Relationships between variables were examined by multiple linear regressions and partial least squares structural equation modeling. Limited nutrition literacy was evident in 46.3% of the HD patients, associated with older age, lower education level, and shorter dialysis vintage. Dietary adherence rate was at 34.9%. Nutrition literacy (*β* = 0.390, *p* < 0.001) was an independent predictor of dietary adherence, mediated by self-efficacy (SIE = 0.186, BC 95% CI 0.110–0.280) and self-management skills (SIE = 0.192, BC 95% CI 0.103–0.304). Thus, nutrition literacy-enhancing strategies targeting self-efficacy and self-management skills should be considered to enhance dietary adherence in the HD population.

## 1. Introduction

Hemodialysis (HD) is the most common renal replacement therapy (RRT) modality for patients with end-stage renal failure (ESRD) [1]. However, a typical 4 h and thrice-weekly HD treatment alone is not adequate to save ESRD patients from nutrition-related problems [2] such as protein energy wasting, mineral bone disorders and electrolyte imbalance [3]. Thus, in addition to dialysis and drug therapies, medical nutrition therapy is deemed as the cornerstone of a successful HD management [4]. However, it depends largely on the patient’s initiative to adhere to the nutrition advices given by healthcare professionals [5]. 

Dietary non-adherence is a significant and pervasive health behavioural issue in the HD population [6], attributed to its complexity and highly restrictive regimen [7]. For instance, the prevalence of dietary non-adherence in HD patients has ranged from 41.1–98.3% [8]. Such problems predispose HD patients to hazardous health complications, including cardiovascular events [9], renal osteodystrophy [10], frequent hospitalisation [11], and increased mortality [12]. To address this issue, understanding factors that associate with dietary adherence behaviour in HD patients is imperative. 

A growing body of evidence has supported the essential role of health literacy on patients’ self-care and adherence behaviours in the HD population [13,14,15,16]. Health literacy is defined as “the skills, knowledge, motivation and the capacity of a person to access, understand, appraise and apply information to make effective decisions about health and health care, and take action” [17]. It has been applied to health behaviour [18], health outcomes [19] and treatment adherence [20]. Now, the health literacy research paradigm has also been extended to the field of nutrition and dietetics [21], leading to the term “nutrition literacy” [22], and well-cited concerning eating practices [23]. 

Given the limited health resources, individuals in low- and middle-incomes countries (LMIC) are prone to inadequate health literacy [24]. Correspondingly, HD patients in LMIC are suffering from limited access to dietitian services attributed to dietitian shortage (20–45%) [25]. Limited nutrition education delivery is likely to affect nutrition literacy, leading to suboptimal dietary adherence in the HD population [14].

Importantly, the health literacy skills framework proposed by Squiers et al. [26] posits a causal pathway that underpins the full continuum of relation from health literacy to health behaviours through mediation. To date, empirical evidence for such a mechanism in the HD population is limited to only one study [15], which showed that disease-specific knowledge was a mediator between health literacy and medical adherence. However, this study did not capture the complete spectrum of health literacy skills, while the statistical approach used in establishing mediation may not have been appropriate [27]. Hence, there is a noteworthy gap pertaining to the theoretical generalizability of this health literacy skills framework [26] to the HD population. Further, the mediating roles of other significant predictors of dietary adherence such as health belief [28,29,30,31] and self-management skills [32,33] remain unveiled. 

Taken together, this study aimed to explore the causal model linking nutrition literacy to dietary adherence in the HD population by extending an established health literacy skills framework [26] with potential mediators using a robust statistical analysis [34]. This is to advance the understanding of relationships between sociodemographic factors, dialysis vintage, nutrition literacy, dietary knowledge, health beliefs, self-management skills and dietary adherence, which can serve as a basis to design intervention strategies to promote dietary adherence in the HD population.

## 2. Materials and Methods 

### 2.1. Study Design and Patient Recruitment

This was a multi-centre, cross-sectional study with multi-ethnic patients recruited from 9 HD centres located in the Klang Valley between February 2019 and July 2019. The flowchart of patient recruitment is provided in the Supplementary Materials (Figure S1). HD patients were considered eligible if they were aged ≥ 18 years, and dialysed thrice weekly ≥ six months. Exclusion criteria were if patients had any physical impairment, present with terminal illness and transfer plan to other RRT option. The selection criteria were based on similar studies [14,35] with some adaptations to the local context and according to expert opinions. A total number of 486 HD patients (83.4%) were found eligible from a total screening population of 583 HD patients. Reasons for exclusion (*n* = 97) were: (i) having been dialysed < six months (*n* = 37), (ii) slurred speech (*n* = 2), (iii) blind (*n* = 4), (iv) frail and severely ill (*n* = 25), (v) history of admission (*n* = 9), (vi) deceased (*n* = 8), (vii) undergone transplantation (*n* = 1), (viii) changed RRT modality (*n* = 2), (ix) transferred out (*n* = 2), (x) stunted growth (*n* = 1), and (xi) involved in oral nutrition supplementation studies (*n* = 6).

Subsequently, 250 patients (sample size determined based on previous literatures [36,37,38,39] with the significant level = 5%, statistical power = 80% and non-response rate = 20%) were selected from 486 eligible patients in accordance with the random list generated by IBM Statistical Package for Social Sciences, SPSS (SPSS Inc., Chicago, IL, USA). All subjects gave their informed consent for inclusion before they participated in the study. The study was conducted in accordance with the Declaration of Helsinki, and the protocol was approved by the Medical Research and Ethics Committee, Ministry of Health, Malaysia (NMRR-18-1514-42126) and the Medical Research Ethics Committee of Universiti Putra Malaysia (JKEUPM-2019-064).

### 2.2. Evaluating Aspects

Recruited patients were assessed for (i) sociodemographic characteristics (i.e., age, gender, ethnicity, marital status, education level, employment status and monthly income), and (ii) dialysis vintage as control variables; (iii) nutrition literacy as independent variable; (iv) dietary adherence as dependent variable; (v) dietary knowledge, (vi) health beliefs of dietary adherence and (vii) self-management skills as mediators.

### 2.3. Questionnaire Development and Testing

#### 2.3.1. Nutrition Literacy

Nutrition literacy of HD patients was assessed with the Dialysis-Specific Nutrition Literacy Scale (DSNLS). Firstly, DSNLS was developed by modifying an existing health literacy questionnaire known as the European Health Literacy Questionnaire (HLS-EU-Q) [40], which has been validated across 6 Asian countries including Malaysia [41]. To date, HLS-EU-Q is the only instrument that has been validated in Malaysia to measure the different spectra of health literacy [42]. 

As per HLS-EU-Q, DSNLS was guided by a conceptual model synthesised by a systematic review of 12 models and 17 definitions of health literacy [17]. Corresponding to Sørensen et al. [17] and Nutbeam (2000) [43], DSNLS measures the patient’s perceived ability to obtain (interactive literacy), understand (functional literacy), appraise and apply (critical literacy) dialysis-related nutrition information. A sample of DSNLS is attached as a supplementary material (Table S1). It consists of 8 items rated from 1 (poor ability) to 3 (good ability). Then, the mean scores of nutrition literacy were transformed into continuous indices [44,45,46,47] using the formula: Index = (Mean − 1) × (100/2), which ranges from 0 to 100, with a higher index denoting greater nutrition literacy. 

Content validation of DSNLS was conducted among six renal healthcare professionals (including nephrologists, dietitians, dialysis nurse and dialysis technician), and resulted in good content validity (S-CVI/Ave = 0.96) [48]. Additionally, the overall DSNLS index shown significant correlation with that of HLS-EU-Q16, with good intra-class coefficients (ICC = 0.792) [49]. In the current study, DSNLS also demonstrated good reliability indicated by internal consistency (Cronbach’s alpha = 0.916), inter-rater reliability (mean weighted kappa = 0.851) and test-retest reliability (ICC = 0.992).

#### 2.3.2. Dietary Adherence

To date, there is no “gold standard” method for assessing dietary adherence in HD patients [50]. Thus, a combination method, using both subjective and indirect measures of dietary adherence, is recommended to enhance data validity [6].

##### Adherence Behaviours

The dietary adherence behaviours of patients were assessed primarily using a validated questionnaire, known as End-Stage Renal Disease Adherence Questionnaire (ESRD-AQ), which has been widely used to study HD patients’ adherence behaviours [51,52,53,54]. The original questionnaire consists of 4 subscales pertaining to dialysis, medication, dietary and fluid adherence, with all four subscales having reported content validity above 0.86 and good reliability (ICCs ≥ 0.83) [55]. In the current study, only the dietary subscale of ESRD-AQ was used. It consists of 8 questions to gauge the patient’s exposure to diet counselling and dietary adherence behaviour. The dietary adherence behaviour was scored by a single question, whereby the scoring system was modified into a continuous rating scale, composed of 0% (none of the time) to 100% (all of the time) [56], with higher scores indicating better adherence. 

##### Dietary Intake 

The ESRD-AQ scores were validated with the estimated nutrients intakes assessed by a 3-day diet record (3DDR) inclusive of one dialysis day, one non-dialysis day and one weekend day [57]. Patients were interviewed about the types of foods and beverages consumed as well as the respective portion sizes, cooking methods, meal timing and meal location. The portion sizes of the foods and beverages were estimated using a set of standardised household measurements to minimise reporting biases. The diet records were analysed using Nutritionist Pro software (version 4.0.0, Axxya System LLC, Stafford, TX, USA) to estimate the individual nutrient intakes. To ensure validity, dietary data were checked for misreporting based on the ratio of reported energy intake to basal metabolic rate (EI: BMR) [58]. 

##### Laboratory Measures

Laboratory values such as pre-dialysis serum phosphate and potassium are the most common parameters used to assess dietary adherence among HD patients [6]. In this study, the patients’ latest biochemical data were collected retrospectively from their medical records and compared with the scores of ESRD-AQ to assess their validity.

#### 2.3.3. Dialysis-Related Dietary Knowledge

A 25-item dialysis-related dietary knowledge questionnaire (DDKQ) was modified from Durose et al. [59], based on the Malaysian Medical Nutrition Therapy Guidelines for Chronic Kidney Disease [60], which was adopted from the National Kidney Foundation K/DOQI Clinical Practice Guidelines for Nutrition in Chronic Renal Failure [57]. Patient knowledge regarding renal diet was probed covering general complications of dietary non-adherence and dietary sources of phosphate, potassium and sodium. A sample questionnaire of the DDKQ is attached as a supplementary material (Table S2). Questions were multiple-choice with an additional “not sure” category to avoid guessing bias. Each correct response was scored “1” while a “0” score was allocated for incorrect answers and “not sure” responses. The knowledge scores were summed and expressed as a percentage of the total scores. This scale had good content validity (S-CVI/Ave = 0.972), internal consistency (Cronbach’s alpha = 0.766), inter-rater reliability (mean weighted kappa = 0.867) and test-retest reliability (ICC = 0.944).

#### 2.3.4. Health Belief

A self-developed questionnaire (dialysis diet-related health belief questionnaire, DDHBQ) was used to assess the patients’ perceptions on the renal diet based on Health Belief Model (HBM). This model has been widely used to predict dietary adherence in HD population over the past decades [28,29,30,31]. DDHBQ consists of 28 items to assess five HBM constructs, namely, (i) perceived benefits, (ii) perceived barrier, (iii) perceived seriousness, (iv) perceived susceptibility and (v) perceived self-efficacy. DDHBQ was developed by modifying validated scales and reviewing qualitative findings from the literature [35,61,62,63,64,65]. Questions that assessed perceived benefits of dietary adherence (7 items) and perceived barriers of dietary adherence (5 items) were developed by modifying the validated scales from the previous studies [61,62] and considering the qualitative findings of previous HD studies [35,63]. Perceived seriousness of sequelae of dietary non-adherence (5 items) and perceived susceptibility to sequelae of dietary non-adherence (5 items) were modified from Welch and colleagues [62]. Perceived self-efficacy of dietary adherence (6 items) was modified from the previously validated Cardiac Diet Self-Efficacy instrument [64,65]. 

Items were scored using a 5-point Likert scale from 1 (strongly disagree/not confident at all) to 5 (strongly agree/very confident). Continuous composite scores [44,45,46,47] were computed for each HBM construct, with higher scores indicating greater measures. A sample questionnaire of the DDHBQ is attached as a supplementary document (Table S3). All constructs in DDHBQ had good content validity indices (S-CVI/Ave = 0.92–1.00) and showed substantial inter-rater reliability (mean weighted kappa = 0.762–0.916) as well as test-retest reliability (ICC = 0.850–0.986). However, except for the perceived barrier (Cronbach’s alpha = 0.324), all HBM constructs achieved acceptable internal consistency (Cronbach’s alpha = 0.719–0.824). This is expected since different types of the barrier were measured in this study, namely (i) knowledge barrier, (ii) limited food choices and (iii) food preference. 

#### 2.3.5. Self-Management Skills

Self-management skills were assessed using a valid and reliable tool known as the Perceived Kidney/Dialysis Self-Management Scale (PKDSMS). It had a reported Cronbach’s alpha of 0.760 in the HD population [66]. Moreover, the scores of PKDSMS were correlated with the self-management behaviours, self-reported health status and serum phosphate in the HD population [66]. PKDSMS consists of 8 items with the Likert scaled responses ranging from 1 (strongly disagree) to 5 (strongly agree). The mean scores from all eight items were transformed into continuous composite scores [44,45,46,47], with higher scores denoting better self-management skills. In the current study, there was a good internally consistent (Cronbach’s alpha = 0.865) and substantial inter-rater reliability (mean weighted kappa = 0.722) and test-retest reliability (ICC = 0.994).

### 2.4. Questionnaire Administration

Before conducting the actual data collection, pre-testing of the questionnaire was performed with 30 HD patients to determine the clarity and reliability of the questionnaire. Subsequently, data collection was carried out with 250 random samples. After giving informed consent, recruited respondents met trained research staff via face-to-face interviews using the finalised semi-structured questionnaire. Interview sessions were divided into two sessions on two consecutive dialysis days to minimise the respondent burden. At the first encounter, patients were interviewed regarding (i) sociodemographic characteristics, (ii) nutrition literacy, (iii) dietary knowledge, and (iv) one-day diet record. At the second encounter, patients were interviewed regarding (v) self-reported dietary adherence, (vi) health beliefs of dietary adherence, (v) self-management skills, and (iv) two-day diet record. Each interview session took about 30 to 40 min to be completed. Other information (e.g., dialysis vintage and laboratory results) was retrieved from medical records.

### 2.5. Measurement Model

Prior to the partial least squares structural equation modeling (PLS-SEM) analysis, the measurement model was evaluated. The measurement model consists of (i) single-item constructs (i.e., limited food choice barrier, dietary adherence and dietary knowledge), (ii) first-order reflective models (i.e., nutrition literacy, perceived benefit, perceived seriousness, perceived susceptibility, perceived self-efficacy, knowledge barrier, food preference barrier and self-management skills) and (iii) second-order reflective-formative models (i.e., perceived barrier). According to Hair et al. (2017) [39], assessment of reflective model involves (1) internal consistency, (2) convergent validity and (3) discriminant validity while formative model involves collinearity assessment and outer weight significance test. Nevertheless, single-item constructs are exempted from assessment [39]. 

### 2.6. Statistical Analyses

Statistical analyses were performed using IBM SPSS version 25.0 and SmartPLS version 3.0. Confirmatory composite analysis (CCA) was used to evaluate the measurement model [67]. Convergent validity and internal consistency were assessed by average variance extracted (AVE ≥ 0.5) and composite reliability (CR ≥ 0.7), respectively. Whereas, divergent validity was determined by the heterotrait–monotrait ratio of correlations (HTMT < 0.85) [68]. Collinearity was assessed using the variance inflation factor (VIF < 5) and outer weight significance test (*t* > 1.96) according to the rule of thumb recommended by Hair et al. (2017) [39]. Items with factor loadings greater than 0.5 were retained in the subsequent statistical analysis. Meanwhile, the structural model was assessed for collinearity issue (VIF < 5), explanatory power (R^2^ ≥ 0.1) and predictive relevance (Q^2^ > 0) [69]. Categorical variables were expressed as frequencies and percentages, whereas continuous variables were presented as mean ± standard deviation. To determine the rates of nutrition literacy and dietary adherence in the HD population, scores of nutrition literacy and dietary adherence were dichotomous (i.e., ≤50 score = limited literacy/non-adherence). Mean scores of nutrition literacy and dietary adherence were compared across sociodemographic factors and dialysis vintage using independent t-test and one-way ANOVA. Relationships between nutrition literacy, dietary knowledge, health belief, self-management skills and dietary adherence were analysed using Pearson’s product-moment correlation. Multiple linear regression (MLR) was used to determine the predictors of nutrition literacy. Hierarchical MLR was used to determine the predictors of dietary adherence by entering variables in an order based on the theoretical model [26]; control variables in block 1; nutrition literacy in block 2; and all potential mediators in block 3. The mediation analysis was performed by PLS-SEM using the bootstrapping method (two-tailed; 5000 resamples) as recommended by Preacher and Hayes [27,70]. Statistical significance was set as 0.05. 

## 3. Results

### 3.1. Assessment of the Measurement Model

The results from the assessment of the measurement model are presented in Table S4 in the Supplementary Materials. All models with reflective indicators exhibited good convergent validity and internal consistency values; nutrition literacy (AVE = 0.615, CR = 0.927), perceived benefit (AVE = 0.540, CR = 0.854), perceived seriousness (AVE = 0.575, CR = 0.844), perceived susceptibility (AVE = 0.537, CR = 0.816), perceived self-efficacy (AVE = 0.526, CR = 0.868), self-management skills (AVE = 0.562, CR = 0.909), knowledge barrier (AVE = 0.861, CR = 0.925) and food preference barrier (AVE = 0.686, CR = 0.813). Divergent validity was also established for all models with reflective indicators (all HTMT ratio < 0.85). Although the indicators of the perceived barrier (formative model) did not have a collinearity issue (VIF < 5), the outer weights of food preference and limited food choices indicators were not statistically significant (*t* ≤ 1.96). However, these indicators were retained due to their conceptual relevance [71] and content validity by means of expert assessment [39].

### 3.2. Patients’ Characteristics

Out of 250 eligible patients approached, 32 patients refused to participate in this study. A final number of 218 patients were recruited into the study (response rate = 87.2%). The patients’ characteristic of this study is shown in Table 1. There were slightly more males (*n* = 116, 53.2%), with an ethnic distribution of 125 Malays (57.4%), 65 Chinese (29.8%) and 28 Indians (12.8%). The mean age of the patients was 54.8 ± 12.8 years, ranging from 18–77 years. The majority of the patients were married (83.9%), completed at least secondary school education (71.5%), unemployed (75.2%), and had monthly incomes less than or equal to RM1000 (55.0%). More than half (51.4%) of the patients have been dialysed for more than 48 months. The dialysis vintage ranged from 6–272 months, with a mean value of 67.2 ± 54.3 months.

### 3.3. Nutrition Literacy

Comparisons of nutritional literary by patient characteristics are presented in Table 2. About 46.3% (101/218) of the HD patients had limited nutrition literacy. On a 100-point scale, the mean index of nutrition literacy was 53.6 ± 31.4. The nutrition literacy index was significantly associated with age (*p* < 0.001), ethnicity (*p* = 0.006), education level (*p* < 0.001), employment status (*p* = 0.014), monthly income (*p* = 0.001), and dialysis vintage (*p* = 0.012). The nutrition literacy index was lower among the older patients; those with a lower level of education and those with a shorter dialysis vintage. Malay patients had significant higher nutrition literacy than Indian patients (58.7 ± 30.1 vs. 39.3 ± 30.0). The nutrition literacy was higher among those who were working and those with monthly income >RM1000.

Based on MLR analysis Table 3, age (*β* = −0.202, *p* = 0.014) and dialysis vintage (*β* = 0.240, *p* < 0.001) were significantly associated with nutrition literacy. Compared to those with college/university level of education, those without formal education (*β* = −0.200, *p* = 0.004), primary education (*β* = −0.443, p < 0.001) and secondary education (*β* = −0.307, *p* < 0.001) had lower levels of nutrition literacy. The model accounted for 29.9% of the variability in nutrition literacy. 

### 3.4. Dietary Adherence

In the current study, the validity of self-reported dietary adherence was strengthened by its significant correlations with serum phosphate (*r* = −0.260, *p* < 0.001) and serum potassium (*r* = −0.184, *p* < 0.001). In addition, self-reported dietary adherence was also negatively correlated with higher dietary intakes of potassium (*r* = −0.234, *p* = 0.001) and phosphorus (*r* = −0.148, *p* = 0.040). No significant correlation was found between self-reported dietary adherence and dietary protein intake (*p* > 0.05).

Comparisons of dietary adherence by patient characteristics are presented in Table 2. The dietary adherence rate based on the patient self-reporting was 34.9%. The mean dietary adherence score was 46.5 ± 27.1 (per 100 scores). Female patients had a significantly higher mean dietary adherence score compared to the males (54.1 ± 24.3 vs. 39.9 ± 27.8, *p* < 0.001). In addition, dietary adherence was also significantly correlated with nutrition literacy (*r* = 0.325, *p* < 0.001), dietary knowledge (*r* = 0.361, *p* < 0.001), perceived benefit (*r* = 0.246, *p* < 0.001), perceived barrier (*r* = −0.369, *p* < 0.001), perceived self-efficacy (*r* = 0.550, *p* < 0.001) and self-management skills (*r* = 0.465, *p* < 0.001).

The results from hierarchical MLR analyses are presented in Table 3 as Blocks 1 to 3 under Model 2. In Block 1, only gender was significant (*β*_female_ = 0.287, *p* < 0.001). In Block 2, nutrition literacy (*β* = 0.390, *p* < 0.001) was significantly associated with dietary adherence. Over and above the sociodemographic variables and dialysis vintage, nutrition literacy contributed an additional 10.7% of variance in dietary adherence (∆R^2^ = 0.107, ∆F (1, 194) = 27.502, *p* < 0.001). With the addition of nutrition literacy, age became significant as well (*β* = 0.246, *p* = 0.005). Female gender (*β* = 0.248, *p* < 0.001) remained significant.

The addition of dietary knowledge, health belief and self-management skills in hierarchical MLR model (Block 3 in Model 2) accounted for an additional 16.5% of the variance in dietary adherence, (∆R^2^ = 0.165, ∆F (7, 187) = 7.504, *p* < 0.001). In the final block of Model 2, only female gender (*β* = 0.177, *p* = 0.005), perceived self-efficacy (*β* = 0.338, *p* < 0.001) and self-management skills (*β* = 0.246, *p* = 0.002) were significant. 

### 3.5. Mediation Analysis

**Figure 1:**
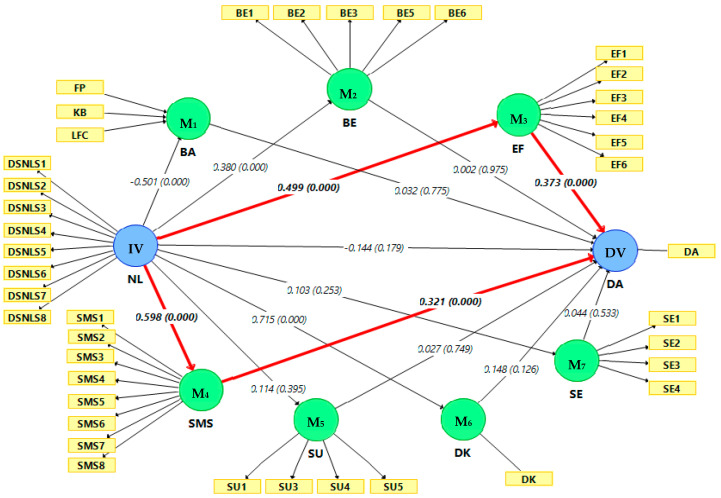
Bootstrapping result for mediation analysis (5000 resamples and 2-tailed). The figure shows the hypothesised mediation paths connecting nutrition literacy to dietary adherence in HD patients. Significant mediation paths are highlighted by red bold lines. Data are presented as path coefficient for the direct effect (*p*-value); NL: Nutrition literacy; DK: Dietary knowledge; BE: Perceived benefit; FP: Food preference; KB: Knowledge barrier; LFC: Limited food choices; BA: Perceived barrier; SE: Perceived seriousness; SU: Perceived susceptibility; EF: Perceived self-efficacy; SMS: Self-management skills; DA: Dietary adherence; IV: Independent variable; DV: Dependent variable; M: Mediator.

In the final block of Model 2 (Table 3), the relationship between nutrition literacy and dietary adherence was attenuated and became non-significant after dietary knowledge, health belief and self-management skills were included into the model, suggesting the presence of mediating effects [72]. Perceived self-efficacy and self-management skills were seen to be the potential mediators between nutrition literacy and dietary adherence. The mediating effects were further examined using the bootstrapping method in PLS-SEM. The results are presented in Table 4 and Figure 1.

Based on the results presented in Table 4, the structural model did not have a collinearity issue (VIF < 5). According to Hair et al. [69], this structural model had moderate predictive relevance (Q^2^ = 0.304) and explanatory power (R^2^ = 0.352). No direct effect was found between nutrition literacy and dietary adherence (*β* = −0.144, *p* = 0.179, *t* = 1.344). With regard to the indirect effect (mediation), only two indirect paths were significant; mediation path 3 (nutrition literacy → perceived self-efficacy → dietary adherence; SIE = 0.186, BC 95% CI 0.110–0.280) and mediation path 4 (nutrition literacy → self-management skills → dietary adherence; SIE = 0.192, BC 95% CI 0.103–0.304). The significant mediation paths are illustrated by red bold lines in Figure 1. The nutrition literacy exerted significant direct positive effects on perceived self-efficacy (*β* = 0.499, *p* < 0.001, *t* = 10.571) and self-management skills (*β* = 0.598, *p* < 0.001, *t* = 14.632, which in turn positively and directly affected dietary adherence, with the path coefficients of (*β* = 0.373, *p* < 0.001, *t* = 4.777) and (*β* = 0.321, *p* < 0.001, *t* = 3.980), respectively. This was consistent with the results of Table 3 (Block 3 in Model 2).

### 3.6. Full Relationship Continuum

**Figure 2:**
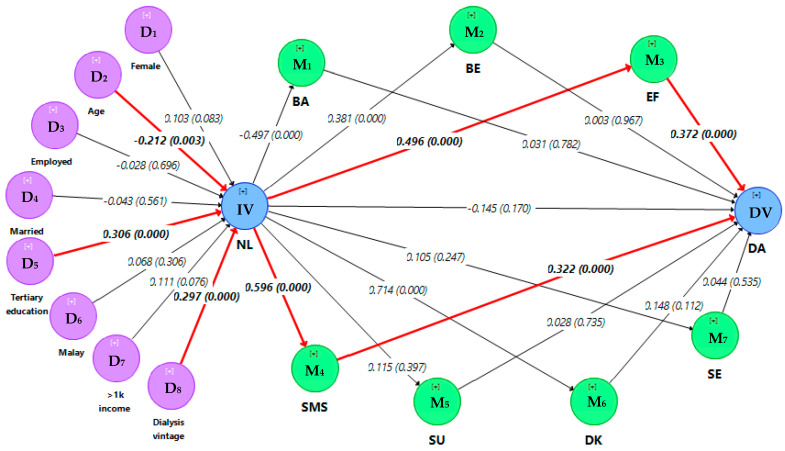
Bootstrapping result for serial mediation analysis (5000 resamples and 2-tailed). The figure shows the full relationship continuum from sociodemographic factors and dialysis vintage to nutrition literacy, patient-related factors and finally dietary adherence in HD patients. Significant mediation paths are highlighted by red bold lines. Data are presented as path coefficient for the direct effect (*p*-value); NL: Nutrition literacy; DK: Dietary knowledge; BE: Perceived benefit; BA: Perceived barrier; SE: Perceived seriousness; SU: Perceived susceptibility; EF: Perceived self-efficacy; SMS: Self-management skills; DA: Dietary adherence; IV: Independent variable; DV: Dependent variable; M: Mediator; D: Determinants.

PLS-SEM analysis for the full relationship continuum further revealed the mediating role of nutrition literacy on the relationships between sociodemographic factors, dialysis vintage and dietary adherence in HD patients. The bootstrapping result for serial mediation is illustrated in Figure 2. The full structural model had moderate predictive relevance (Q^2^ = 0.304) and explanatory power (R^2^ = 0.328). Six serial mediation paths that explained the full relationship continuum were identified (Table 5); serial mediation path 1 (age → nutrition literacy → perceived self-efficacy → dietary adherence; SIE = −0.039, BC 95% CI −0.078–−0.014), serial mediation path 2 (education → nutrition literacy → perceived self-efficacy → dietary adherence; SIE = 0.057, BC 95% CI 0.031−0.102), serial mediation path 3 (dialysis vintage → nutrition literacy → perceived self-efficacy → dietary adherence; SIE = 0.055, BC 95% CI 0.032−0.095), serial mediation path 4 (age → nutrition literacy → self-management skills → dietary adherence; SIE = −0.041, BC 95% CI −0.084–−0.013), serial mediation path 5 (education → nutrition literacy → self-management skills → dietary adherence; SIE = 0.059, BC 95% CI 0.026−0.110), serial mediation path 6 (dialysis vintage → nutrition literacy → self-management skills→ dietary adherence; SIE = 0.057, BC 95% CI 0.027−0.102).

## 4. Discussion

To the best of our knowledge, this is the first study to address dialysis-specific nutrition-related health literacy among HD patients. In the current study, a greater proportion of HD patients were found to exhibit limited nutrition literacy compared to limited health literacy as reported in earlier studies [73,74,75]. This might imply that HD patients are confronted with a greater difficulty in obtaining, understanding and processing nutrition information compared to general health information. Constraints of the healthcare system in dialysis settings are the probable reasons for such discrepancies [32]. Lack of dietetic services in the HD setting is a critical problem reported in Malaysia and other Asian countries [76,77]. As such, nephrologists and nurses have become the primary sources of dietary information for HD patients [76,78]. However, due to time constraints [79], nutrition education might not always be their priority. For instance, the majority of the patients (44.5%) in this study claimed that diet counselling from health professionals was rarely provided. In addition, the quality of patient nutrition education is also likely to be undermined by nutrition knowledge incompetency among healthcare professionals [78]. Therefore, the provision of dietitians in dialysis centres remains advocated for successful nutrition management [80].

Due to the healthcare constraints, the patient’s initiative to seek nutrition information is imperative. Nonetheless, about 46.3% of the patients in this study were found to have limited interactive literacy skills, probably due to language barrier [81], which hinders them from acquiring nutrition information from health professionals. Besides the language barrier, previous studies found that patients with limited health literacy are often reluctant to seek assistance from healthcare professionals due to shame [82]. They feel embarrassed to admit their difficulty in understanding health information. As such, the stigma of low health literacy may discourage patients from disclosing their problems to healthcare professionals. In contrast, HD patients in the present study had greater accessibility to nutrition information from non-medical sources such as the internet, family members or even their peers. However, the credibility of these sources is questionable [83]. Without adequate critical literacy skills to appraise the content relevance and accuracy, HD patients with limited nutrition literacy are susceptible to obtaining misleading nutrition information. 

Given the high prevalence of limited nutrition literacy among HD patients, healthcare professionals should take an active role in screening those at-risk patients for more intensive support [84]. However, detecting limited nutrition literacy can be challenging in the clinical setting as patients are likely to hide their literacy problems associated with stigma as discussed above [82]. For this reason, the current study has revealed the determinants of nutrition literacy to help healthcare practitioners to identify HD patients at risk of limited nutrition literacy. Based on the findings, healthcare professionals should pay more attention and offer additional support as required when dealing with HD patients who are older, have lower education level and are new to dialysis. Older HD patients are vulnerable to limited nutrition literacy attributed to cognitive, physical and psychosocial impairments [85,86]. Consistent with earlier studies [73,74,75], nutrition literacy was associated with a lower education level in this study. This is expected since the functional literacy skills that allow individuals to understand the health information are highly associated with basic learning skills such as reading, writing and calculating [43]. Regardless of the age factor, this study found that patients with longer dialysis vintage were associated with greater nutrition literacy. This implies that HD patients might develop nutrition literacy skills and accumulate knowledge and experiences over time. In addition to these determinants, the clinical “red flag” for limited health literacy proposed by the American Medical Association Foundation [87] can also be a useful tool to help healthcare professionals to identify patients with limited health literacy in the clinical setting [88].

The dietary non-adherence rate found in this study is consistent with the findings of a systematic review [50]. As concluded by the earlier study [14], nutrition literacy was a significant predictor of dietary adherence in HD patients. To explain this, health inequality is deemed as a probable reason [89]. Under the same healthcare system and resources, patients with limited nutrition literacy are expected to experience greater difficulty in seeking, comprehending and assimilating dietary information, leading to unintentional dietary non-adherence [90,91]. Importantly, nutrition literacy was found as a stronger predictor of dietary adherence compared to sociodemographic factors in the current study. This is in line with the position of the American Medical Association on health literacy [92]. Not only that, the influence of nutrition literacy on dietary adherence is beyond its predictive role. In the present study, nutrition literacy was also identified as a mediator that linked sociodemographic factors and dialysis vintage to dietary adherence. This is in tune with the previous studies pertaining to the mediating role of health literacy on the relationship between sociodemographic factors and health [93,94]. It also explains why the relationship between health literacy and dietary adherence is more prominent among patients with lower socio-economic status [14].

To our knowledge, this study is probably the first study to model the full relationship continuum between health literacy and dietary adherence in the HD populations as posited by theoretical frameworks [16,95]. We provide the empirical evidence to support the hypothesis that HD patients with adequate nutrition literacy have greater confidence to follow dietary instructions and higher chance of achieving health goals which subsequently facilitates dietary adherence behaviour. In other words, an individual’s self-efficacy and self-management skills explain comparably how adequate nutrition literacy leads to better dietary adherence in HD patients. Self-efficacy and self-management skills have been recognised as the core factors in promoting behavioural change in the nutritional management of HD patients, and health literacy has been recognised as a requisite factor in this relationship [79].

Although perceived risk has been found to be associated with limited health literacy [96] and dietary adherence in CKD patients [63], we could not find any significant association in this study. This could be due to the fact that the health complications of dietary non-adherence are generally long-term events and often have subtle symptoms. In addition, most of our patients also had limited skills in accessing, understanding and interpreting their laboratory results. Therefore, our patients might tend to underestimate the seriousness and susceptibility to health complications attributed to dietary non-adherence.

The relationship between knowledge and adherence behaviours is a long-debated topic. Yet, the results are mixed as to whether increased knowledge leads to better adherence [97]. The role of knowledge on dietary adherence behaviour in the HD population is also in the same vein [6,15,35]. In this study, dietary knowledge was neither a predictor of dietary adherence nor a mediator of the relationship between nutrition literacy and dietary adherence. Although significant correlations were observed between dietary knowledge with nutrition literacy and dietary adherence, they did not hold out in the multivariable analyses. Thus, we conclude that increasing knowledge alone might not be sufficient to promote dietary adherence in the HD population. 

To explain this, intentional non-adherence and patients’ health belief are brought to the table. Patients with high nutrition literacy skills, and presumably knowledgeable about renal diet, are likely to exhibit intentional non-adherence behaviours [91]. For instance, out of 117 patients who had adequate nutrition literacy in the current study, about 57.3% (67/117) might advertently not adhere to dietary instructions. This is because, despite having dietary knowledge, patients with adequate health literacy may confront other types of barriers such as food preference and limited food choices [35,63,71], which prevent them from adhering to the dietary recommendations. It is important to acknowledge that intentional and unintentional non-adherence are two different entities. As per the proverb “different strokes for different folks”, different intervention strategies are needed when dealing with intentional and unintentional non-adherence [90]. Enhancing patients’ knowledge about renal diet should remain the primary objective in nutrition education, especially for those with low nutrition literacy and dietary knowledge. In addition, to enhance understanding of nutrition information among patients with limited nutrition literacy, strategies suggested by the Health Literacy Universal Precautions Toolkit [98] may be adopted. This includes the teach-back method, which requires patients to repeat the instructions given to ensure that they adequately understand the delivered nutrition information. Besides, healthcare practitioners should consciously avoid medical jargon and seek feedback from patients during the consultation. Likewise, infographics should be incorporated into education materials to foster the patients’ reading comprehension. Conversely, for intentional non-adherence, shared decision making is expected to better align the nutrition care plan with the patient’s condition, eating habits and lifestyle to enhance their self-efficacy and self-management skills in adhering to the dietary regimen [99,100]. Additionally, studies reported that intentional non-adherence is sometimes accompanied by idiosyncratic beliefs [101]. Thus, assessment and correction of false beliefs are crucial in encouraging behavioural change when dealing with intentional non-adherence.

All in all, the major implication of this study is that information on the nutrition literacy competencies of a dialysis patient is important in making a difference to patient-centered care for promoting dietary adherence behaviours. We provide the empirical evidence to support the theoretical generalisation of the health literacy skills framework proposed by Squires et al. [26] for the HD population. The proposed model (Supplementary Figure S2) is expected to foster the understanding pertaining to the interrelationships between sociodemographic factors, dialysis vintage, nutrition literacy, dietary knowledge, health belief, self-management skills and dietary adherence behaviours in the HD population. The external validity of the findings was supported by the use of a probability sampling method, a good response rate and good sample representativeness in term of ethnicity distribution. Future research is needed to verify the validity and generalisability of this model. The next research direction is to examine the impact of limited nutrition literacy on nutritional outcomes attributed to dietary non-adherence among HD patients.

The major limitation of this questionnaire-based study was the potential data collection bias inherent to patient’s self-report. Thus, it is susceptible to interviewer bias (i.e., preconceived judgment) [102] and common method bias (i.e., social desirability bias) [103]. Nevertheless, the data quality was reassured by good inter-rater and test-retest reliability, as well as validation with the objective measurements (i.e., laboratory results). Secondly, the nutrition literacy, dietary knowledge and health beliefs of HD patients were assessed by self-developed scales. Although the validity and reliability of these scales have been established in this study, replication studies are necessary to verify its psychometric validity. Lastly, the findings of this cross-sectional study cannot be used to establish causality. Rather, our findings provide empirical evidence to support the causal assumption on how nutrition literacy influences dietary adherence in HD patients.

## 5. Conclusions

Limited nutrition literacy and dietary non-adherence are prevalent in the HD population. HD patients of older age, lower education and shorter dialysis vintage are at risk of limited nutrition literacy. Nutrition literacy is not only an independent predictor of dietary adherence, but it is also a mediator of the relationships between sociodemographic factors, dialysis vintage and dietary adherence through self-efficacy and self-management skills. Therefore, patients’ nutrition literacy serves as an X-factor in promoting dietary adherence among HD patients. Nutrition literacy-enhancing strategies should target self-efficacy and self-management skill development in the HD population which plausibly will enhance dietary adherence.

## Figures and Tables

**Table 1:** Sociodemographic Profile of the Respondents (*n* = 218).

Variables	*n* (%)	Mean (SD)	Range
Gender			
Male	116 (53.2)		
Female	102 (46.8)		
Age (years)		54.8 (12.8)	18–77
18–30	12 (5.5)		
31–40	24 (11.0)		
41–50	32 (14.7)		
51–60	61 (28.0)		
>60	89 (40.8)		
Ethnicity			
Malay	125 (57.4)		
Chinese	65 (29.8)		
Indian	28 (12.8)		
Marital Status			
Single	25 (11.5)		
Married	183 (83.9)		
Divorced	10 (4.6)		
Education Level			
None	8 (3.7)		
Primary	54 (24.8)		
Secondary	101 (46.3)		
Tertiary	55 (25.2)		
Employment Status			
Working	54 (24.8)		
Not working	164 (75.2)		
Monthly Income			
≤RM1000	120 (55.0)		
>RM1000	98 (45.0)		
Dialysis Vintage (months)		67.2 (54.3)	6–272
<12	17 (7.8)		
12–48	89 (40.8)		
>48	112 (51.4)		

**Table 2:** Comparisons of Nutrition Literacy and Dietary Adherence across Patients’ Characteristics (*n* = 218).

Variables	Nutrition Literacy	*p*-Value	Dietary Adherence	*p*-Value
Gender		0.280		<0.001
Male	51.5 ± 31.5		39.9 ± 27.8	
Female	56.1 ± 31.3		54.1 ± 24.3	
Age (years)		<0.001		0.907
18–30	60.0 ± 28.2		39.6 ± 22.7	
31–40	75.1 ± 29.9 ^ab^		48.8 ± 23.1	
41–50	64.8 ± 28.4 ^c^		45.9 ± 29.3	
51–60	53.3 ± 30.8 ^a^		47.4 ± 27.8	
>60	43.6 ± 30.0 ^bc^		46.5 ± 27.7	
Ethnicity		0.006		0.428
Malay	58.7 ± 30.1 ^a^		48.5 ± 27.6	
Chinese	50.0 ± 32.5		43.2 ± 26.3	
Indian	39.3 ± 30.0 ^a^		45.2 ± 27.0	
Marital Status		0.244		0.521
Single	62.3 ± 33.5		43.4 ± 19.2	
Married	53.0 ± 30.7		46.5 ± 28.0	
Divorced	44.4 ± 37.4		55.0 ± 27.1	
Education Level		<0.001		0.099
None	34.1 ± 20.2 ^a^		60.6 ± 24.3	
Primary	37.4 ± 26.2 ^bc^		44.7 ± 26.3	
Secondary	53.2 ± 33.1 ^bd^		43.3 ± 27.8	
Tertiary	73.2 ± 22.5 ^acd^		52.2 ± 26.1	
Employment Status		0.014		0.946
Working	62.8 ± 30.4		46.3 ± 28.7	
Not working	50.6 ± 31.3		46.6 ± 26.7	
Monthly Income		0.001		0.222
≤RM1000	47.5 ± 31.1		48.5 ± 26.5	
>RM1000	61.2 ± 30.3		44.0 ± 27.8	
Dialysis Vintage (months)		0.012		0.602
<12	34.9 ± 26.6 ^a^		43.0 ± 30.4	
12–48	50.8 ± 30.9		47.6 ± 24.4	
>48	58.8 ± 31.4 ^a^		47.5 ± 27.0	

Data are expressed as mean ± standard deviation; Data were analysed using independent *t*-test and one-way ANOVA; data sharing the same superscript are significantly different from each other.

**Table 3:** Predictors of Nutrition Literacy and Dietary Adherence in HD Patients (*n* = 218).

Variables	Model 1		Model 2					
			Block 1		Block 2		Block 3	
	*β*	R^2^	*β*	R^2^	*β*	R^2^	*β*	R^2^
		0.299		0.140		0.247		0.412
Age	−0.202 *		0.167		0.246 **		0.132	
Gender ^a^								
Female	0.101		0.287 ***		0.248 ***		0.177 **	
Ethnicity ^b^								
Chinese	0.054		−0.127		−0.148		−0.109	
Indian	−0.096		0.002		0.035		−0.001	
Marital Status ^c^								
Married	0.039		−0.065		−0.080		−0.073	
Divorced	−0.001		0.058		0.058		0.065	
Education Level ^d^								
None	−0.200 **		−0.011		0.067		0.033	
Primary	−0.443 ***		−0.185		−0.013		−0.034	
Secondary	−0.307 ***		−0.201		−0.081		−0.094	
Employment ^e^ Status								
Working	−0.027		0.136		0.147		0.038	
Monthly Income ^f^								
≤RM1000	−0.030		0.135		0.147		0.040	
Dialysis Vintage	0.240 ***		0.097		0.004		0.015	
Nutrition Literacy					0.390 ***		−0.043	
Dietary Knowledge							0.105	
Perceived Benefit							0.016	
Perceived Barrier							−0.011	
Perceived Seriousness							0.024	
Perceived Susceptibility							0.001	
Perceived Self-Efficacy							0.338 ***	
Self-Management Skills							0.246 **	

Reference group: ^a^ Male, ^b^ Malay, ^c^ Single, ^d^ Tertiary education, ^e^ Not working, ^f^ Income > RM1000. * *p* < 0.05, ** *p* < 0.01, *** *p* < 0.001. Model 1: Multiple Regression Model for Nutrition Literacy; Model 2: Hierarchical Multiple Regression Model for Dietary Adherence. Block 1: Sociodemographic Factors and Dialysis Vintage (control variables)^,^ Block 2: Block 1 + Nutrition Literacy. Block 3: Block 2 + Potential Mediators (Dietary Knowledge, Health Belief, and Self-Management Skills).

**Table 4:** Bootstrapping Result for Direct and Indirect Effects (*n* = 218).

**Direct Effects**	**Path**	***t* (>1.96)**	**BC 95% CI**		***f*^2^**	**VIF** **(<5)**	**R^2^** **(≥0.1)**	**Q^2^** **(>0)**
			**LB**	**UB**				
NL → DA	−0.144	1.344	−0.347	0.071	0.011	2.845	0.352	0.304
DK → DA	0.148	1.532			0.013	2.579		
BE → DA	0.002	0.031			0.000	1.561		
BA → DA	0.032	0.285			0.001	1.729		
SE → DA	0.044	0.642			0.002	1.271		
SU → DA	0.027	0.319			0.001	1.131		
EF → DA	0.373 ***	4.777			0.121	1.776		
SMS → DA	0.321 ***	3.980			0.088	1.817		
NL → DK	0.715 ***	24.640	0.657	0.769	1.048	1.000	0.512	0.508
NL → BE	0.380 ***	6.284	0.267	0.503	0.169	1.000	0.144	0.071
NL → BA	−0.501 ***	7.836	−0.606	−0.375	0.335	1.000	0.251	0.074
NL → SE	0.103	1.144	−0.127	0.254	0.011	1.000	0.011	0.004
NL → SU	0.114	0.850	−0.226	0.264	0.013	1.000	0.013	−0.010
NL → EF	0.499 ***	10.571	0.405	0.593	0.331	1.000	0.249	0.114
NL → SMS	0.598 ***	14.632	0.515	0.678	0.556	1.000	0.357	0.189
**Indirect Effects**	**SIE**	***t* (>1.96)**	**BC 95% CI**		**VAF (%)**			
			**LB**	**UB**				
(1) NL → BA → DA	−0.016	0.291	−0.147	0.076	4.8			
(2) NL → BE→ DA	0.001	0.030	−0.055	0.052	0.3			
(3) NA → EF → DA	0.186 ***	4.406	0.110	0.280	55.9			
(4) NL → SMS → DA	0.192 ***	3.840	0.103	0.304	57.7			
(5) NL → SU → DA	0.003	0.235	−0.026	0.026	0.9			
(6) NL → DK → DA	0.106	1.577	−0.022	0.246	31.8			
(7) NL → SE → DA	0.005	0.400	−0.021	0.027	1.5			

Analysed using Partial Least Squares Structural Equation Modeling (bootstrapping at 5000 resamples, 2-tailed); *** *p* < 0.001; BC 95% CI: Bias-Corrected 95% Confidence Interval, LB: Lower bound, UB: Upper bound; NL: Nutrition literacy; DK: Dietary knowledge; BE: Perceived benefit; BA: Perceived barrier; SE: Perceived seriousness; SU: Perceived susceptibility; EF: Perceived self-efficacy; SMS: Self-management skills; DA: Dietary adherence; SIE: Specific Indirect Effect, VAF: Variance accounted for; VIF: Inner model inflation factor.

**Table 5:** Bootstrapping Result for Serial Mediation (*n* = 218).

Serial Mediation Paths	SIE	*t* (>1.96)	BC 95% CI		VAF (%)
			LB	UB	
(1) Age → NL → EF → DA	−0.039 *	2.489	−0.078	−0.014	55.7
(2) Education → NL → EF→ DA	0.057 **	3.286	0.031	0.102	56.4
(3) Dialysis vintage → NL → EF → DA	0.055 ***	3.567	0.032	0.095	56.1
(4) Age → NL → SMS → DA	−0.041 *	2.332	−0.084	−0.013	58.6
(5) Education → NL → SMS → DA	0.059 **	2.864	0.026	0.110	58.4
(6) Dialysis vintage → NL → SMS → DA	0.057 **	3.082	0.027	0.102	58.2

Note: Only significant paths for the full relationship continuum were presented, analysed using Partial Least Squares Structural Equation Modeling (bootstrapping at 5000 resamples, two-tailed); * *p* < 0.05, ** *p* < 0.01, *** *p* < 0.001; BC 95% CI: Bias-Corrected 95% Confidence Interval, LB: Lower bound, UB: Upper bound; NL: Nutrition literacy; EF: Perceived self-efficacy; SMS: Self-management skills; DA: Dietary adherence; SIE: Specific Indirect Effect, VAF: Variance accounted for.

## Supplementary Materials

The following are available online at https://www.mdpi.com/1660-4601/17/20/7479/s1, Table S1: Dialysis-Specific Nutrition Literacy Scale (DSNLS), Table S2: Dialysis-Related Diet Knowledge Questionnaire (DDKQ), Table S3: Dialysis Diet-Related Health Belief Questionnaire (DDHBQ), Table S4: Results of Confirmatory Composite Analysis for Measurement Model Assessment, Figure S1: Flowchart of Patient Recruitment, Figure S2: Established Casual Model Linking Nutrition Literary to Dietary Adherence in HD Patients.

## References

[1] United States Renal Data System (2018). USRDS Annual Data Report: Epidemiology of Kidney Disease in the United States.

[2] Achinger S.G., Ayus J.C. (2005). The role of daily dialysis in the control of hyperphosphatemia. Kidney Int..

[3] Himmelfarb J., Ikizler T.A. (2010). Hemodialysis. N. Engl. J. Med..

[4] Kopple J.D., Fouque D. (2018). Pro: The rationale for dietary therapy for patients with advanced chronic kidney disease. Nephrol. Dial. Transplant..

[5] Curtin R.B., Mapes D., Schatell D., Burrows-Hudson S. (2005). Self-management in patients with end stage renal disease: Exploring domains and dimensions. Nephrol. Nurs. J..

[6] Lambert K., Mullan J., Mansfield K.J. (2017). An integrative review of the methodology and findings regarding dietary adherence in end stage kidney disease. BMC Nephrol..

[7] Biruete A., Jeong J.H., Barnes J.L., Wilund K.R. (2017). Modified Nutritional Recommendations to Improve Dietary Patterns and Outcomes in Hemodialysis Patients. J. Ren. Nutr..

[8] Gebrie M.H., Ford J. (2019). Depressive symptoms and dietary non-adherence among end stage renal disease patients undergoing hemodialysis therapy: Systematic review. BMC Nephrol..

[9] Luo J., Brunelli S.M., Jensen D.E., Yang A. (2015). Association between Serum Potassium and Outcomes in Patients with Reduced Kidney Function. Clin. J. Am. Soc. Nephrol..

[10] Young E.W., Akiba T., Albert J.M., McCarthy J.T., Kerr P.G., Mendelssohn D.C., Jadoul M. (2004). Magnitude and impact of abnormal mineral metabolism in hemodialysis patients in the dialysis outcomes and practice patterns study (DOPPS). Am. J. Kidney Dis..

[11] Saran R., Bragg-Gresham J.L., Rayner H.C., Goodkin D.A., Keen M.L., Van Dijk P.C., Kurokawa K., Piera L., Saito A., Fukuhara S. (2003). Nonadherence in hemodialysis: Associations with mortality, hospitalization, and practice patterns in the DOPPS. Kidney Int..

[12] Tentori F., Blayney M.J., Albert J.M., Gillespie B.W., Kerr P.G., Bommer J., Young E.W., Akizawa T., Akiba T., Pisoni R.L. (2008). Mortality Risk for Dialysis Patients With Different Levels of Serum Calcium, Phosphorus, and PTH: The Dialysis Outcomes and Practice Patterns Study (DOPPS). Am. J. Kidney Dis..

[13] Green J.A., Mor M.K., Shields A.M., Sevick M.A., Arnold R.M., Palevsky P.M., Fine M.J., Weisbord S.D. (2013). Associations of Health Literacy With Dialysis Adherence and Health Resource Utilization in Patients Receiving Maintenance Hemodialysis. Am. J. Kidney Dis..

[14] Skoumalova I., Kolarcik P., Geckova A.M., Rosenberger J., Majernikova M., Klein D., Van Dijk J.P., Reijneveld S.A. (2019). Is Health Literacy of Dialyzed Patients Related to Their Adherence to Dietary and Fluid Intake Recommendations?. Int. J. Environ. Res. Public Health.

[15] Qobadi M., Besharat M.A., Rostami R., Rahiminezhad A. (2015). Health Literacy and Medical Adherence in Hemodialysis Patients: The Mediating Role of Disease-Specific Knowledge. Thrita.

[16] Devraj R., Gordon E.J. (2009). Health Literacy and Kidney Disease: Toward a New Line of Research. Am. J. Kidney Dis..

[17] Sørensen K., Broucke S.V.D., Fullam J., Doyle G., Pelikan J.M., Słońska Z.A., Brand H., (HLS-EU) Consortium Health Literacy Project European (2012). Health literacy and public health: A systematic review and integration of definitions and models. BMC Public Health.

[18] Aaby A., Friis K., Christensen B., Rowlands G., Maindal H.T. (2017). Health literacy is associated with health behaviour and self-reported health: A large population-based study in individuals with cardiovascular disease. Eur. J. Prev. Cardiol..

[19] Berkman N.D., Sheridan S.L., Donahue K.E., Halpern D.J., Crotty K. (2011). Low Health Literacy and Health Outcomes: An Updated Systematic Review. Ann. Intern. Med..

[20] Miller T.A. (2016). Health literacy and adherence to medical treatment in chronic and acute illness: A meta-analysis. Patient Educ. Couns..

[21] Carbone E.T., Zoellner J.M. (2012). Nutrition and Health Literacy: A Systematic Review to Inform Nutrition Research and Practice. J. Acad. Nutr. Diet..

[22] Velardo S. (2015). The Nuances of Health Literacy, Nutrition Literacy, and Food Literacy. J. Nutr. Educ. Behav..

[23] Cullen T., Hatch J., Martin W., Higgins J.W., Sheppard R. (2015). Food Literacy: Definition and Framework for Action. Can. J. Diet. Pr. Res..

[24] World Health Organization Health literacy toolkit for low- and Middle-Income Countries: A Series of Information Sheets to Empower Communities and Strengthen Health Systems. https://apps.who.int/iris/handle/10665/205244.

[25] Bello A.K., Levin A., Lunney M., Osman M.A., Ye F., Ashuntantang G., Bellorin-Font E., Benghanem G.M., Ghnaimat M., Harden P. (2019). Global Kidney Health Atlas: A Report by the International Society of Nephrology on the Global Burden of End-Stage Kidney Disease and Capacity for Kidney Replacement Therapy and Conservative Care across World Countries and Regions.

[26] Squiers L., Peinado S., Berkman N., Boudewyns V., McCormack L. (2012). The Health Literacy Skills Framework. J. Health Commun..

[27] Hayes A.F. (2009). Beyond Baron and Kenney: Statistical Mediation Analysis in the New Millennium. Commun. Monogr..

[28] Elliott J.O., Ortman C., Almaani S., Lee Y.H., Jordan K. (2015). Understanding the Associations Between Modifying Factors, Individual Health Beliefs, and Hemodialysis Patients’ Adherence to a Low-Phosphorus Diet. J. Ren. Nutr..

[29] Lee S.-H., Molassiotis A. (2002). Dietary and fluid compliance in Chinese hemodialysis patients. Int. J. Nurs. Stud..

[30] Agondi R.D.F., Gallani M.C.B.J., Rodrigues R.C., Cornélio M.E. (2011). Relationship Between Beliefs Regarding a Low Salt Diet in Chronic Renal Failure Patients on Dialysis. J. Ren. Nutr..

[31] Thomas L.K., Sargent R.G., Michels P.C., Richter D.L., Valois R.F., Moore C.G. (2001). Identification of the factors associated with compliance to therapeutic diets in older adults with end stage renal disease. J. Ren. Nutr..

[32] Narva A., Norton J.M., Boulware L.E. (2015). Educating Patients about CKD: The Path to Self-Management and Patient-Centered Care. Clin. J. Am. Soc. Nephrol..

[33] Griva K., Nandakumar M., Ng J.-A.H., Lam K.F., McBain H., Newman S. (2018). Hemodialysis Self-management Intervention Randomized Trial (HED-SMART): A Practical Low-Intensity Intervention to Improve Adherence and Clinical Markers in Patients Receiving Hemodialysis. Am. J. Kidney Dis..

[34] Sarstedt M., Hair J.J.F., Nitzl C., Ringle C.M., Howard M.C. (2020). Beyond a tandem analysis of SEM and PROCESS: Use of PLS-SEM for mediation analyses!. Int. J. Mark. Res..

[35] Chan Y.M., Zalilah M.S., Hii S.Z. (2012). Determinants of Compliance Behaviours among Patients Undergoing Hemodialysis in Malaysia. PLoS ONE.

[36] Kline R. (2016). Principles and Practice of Structural Equation Modeling.

[37] Kock N., Hadaya P. (2016). Minimum sample size estimation in PLS-SEM: The inverse square root and gamma-exponential methods. Inf. Syst. J..

[38] Cohen J. (1992). A power primer. Psychol. Bull..

[39] Hair J.F., Hult G.T., Ringle C.M., Sarstedt M. (2017). A Primer on Partial Least Squares Structural Equation Modeling (PLS-SEM).

[40] Pelikan J.M., Rothlin F., Boltzmann K. Measuring comprehensive health literacy in general population: Validation of instrument, indices and scales of the HLS-EU study. Proceedings of the 6th Annual Health Literacy Research Conference.

[41] Duong T.V., Aringazina A., Baisunova G., Nurjanah, Pham T.V., Pham K.M., Truong T.Q., Nguyen K.T., Myint Oo W., Mohamad E. (2017). Measuring health literacy in Asia: Validation of the HLS-EU-Q47 survey tool in six Asian countries. J. Epidemiol..

[42] Abdullah A., Liew S.M., Salim H.S., Ng C.J., Chinna K. (2020). Health Literacy Research in Malaysia: A Scoping Review. Sains Malays..

[43] Nutbeam D. (2000). Health literacy as a public health goal: A challenge for contemporary health education and communication strategies into the 21st century. Health Promot. Int..

[44] Subedi B.P. (2016). Using Likert Type Data in Social Science Research: Confusion, Issues and Challenges. Int. J. Comtemporary Appl. Sci..

[45] Joshi A., Kale S., Chandel S., Pal D. (2015). Likert Scale: Explored and Explained. Br. J. Appl. Sci. Technol..

[46] Boone H.N., Boone D.A. (2012). Analyzing Likert Data. J. Ext..

[47] Carifio J., Perla R.J. (2007). Ten Common Misunderstandings, Misconceptions, Persistent Myths and Urban Legends about Likert Scales and Likert Response Formats and their Antidotes. J. Soc. Sci..

[48] Polit D.F., Beck C.T. (2006). The content validity index: Are you sure you know what’s being reported? critique and recommendations. Res. Nurs. Health.

[49] Koo T.K., Li M.Y. (2016). A Guideline of Selecting and Reporting Intraclass Correlation Coefficients for Reliability Research. J. Chiropr. Med..

[50] Oquendo L.G., Asencio J.M.M., Nieves C.B.D.L. (2017). Contributing factors for therapeutic diet adherence in patients receiving haemodialysis treatment: An integrative review. J. Clin. Nurs..

[51] Naalweh K.S., Barakat M.A., Sweileh M.W., Al-Jabi S.W., Sweileh W.M., Zyoud S.H. (2017). Treatment adherence and perception in patients on maintenance hemodialysis: A cross—Sectional study from Palestine. BMC Nephrol..

[52] Daniels G.B., Robinson J.R., Walker C.A. (2018). Adherence to Treatment by African Americans Undergoing Hemodialysis. Nephrol. Nurs. J..

[53] Suganthi S., Arjunan P., Geetha P. (2019). Assess the Illness Perception and Treatment Adherence among Patients with End-Stage Renal Disease. Iran. J. Nurs. Midwifery Res..

[54] Opiyo R.O., Nyasulu P.S., Olenja J., Zunza M., Nguyen K.A., Bukania Z., Nabakwe E., Mbogo A., Were A.O. (2019). Factors associated with adherence to dietary prescription among adult patients with chronic kidney disease on hemodialysis in national referral hospitals in Kenya: A mixed-methods survey. Ren. Replace. Ther..

[55] Kim Y., Evangelista L.S., Phillips L.R., Pavlish C., Kopple J.D. (2010). The End-Stage Renal Disease Adherence Questionnaire (ESRD-AQ): Testing the psychometric properties in patients receiving in-center hemodialysis. Nephrol. Nurs. J..

[56] Wu H., Leung S.-O. (2017). Can Likert Scales be Treated as Interval Scales?—A Simulation Study. J. Soc. Serv. Res..

[57] Kopple J.D. (2001). National kidney foundation K/DOQI clinical practice guidelines for nutrition in chronic renal failure. Am. J. Kidney Dis..

[58] E Black A. (2000). Critical evaluation of energy intake using the Goldberg cut-off for energy intake:basal metabolic rate. A practical guide to its calculation, use and limitations. Int. J. Obes..

[59] DuRose C.L., Holdsworth M., Watson V., Przygrodzka F. (2004). Knowledge of dietary restrictions and the medical consequences of noncompliance by patients on hemodialysis are not predictive of dietary compliance. J. Am. Diet. Assoc..

[60] Medical Nutrition Therapy Guidelines for Chronic Kidney Disease Working Group Committee Medical Nutrition Therapy Guidelines for Chronic Kidney Disease: Malaysian Dietitians’ Association. http://storage.unitedwebnetwork.com/files/290/8a1062a0d440dde5c9b5aa3317bb9e89.pdf.

[61] Welch J.L., Bennett S.J., Delp R.L., Agarwal R. (2006). Benefits of and Barriers to Dietary Sodium Adherence. West. J. Nurs. Res..

[62] Welch J.L., Perkins S.M., Evans J.D., Bajpai S. (2003). Differences in perceptions by stage of fluid adherence. J. Ren. Nutr..

[63] Hong L.I., Wang W., Chan E.Y., Mohamed F., Chen H.C. (2017). Dietary and fluid restriction perceptions of patients undergoing haemodialysis: An exploratory study. J. Clin. Nurs..

[64] Hickey M.L., Owen S.V., Froman R.D. (1992). Instrument development: Cardiac diet and exercise self-efficacy. Nurs. Res..

[65] Welch J.L., Astroth K.S., Perkins S.M., Johnson C.S., Connelly K., Siek K.A., Jones J., Scott L.L. (2013). Using a mobile application to self-monitor diet and fluid intake among adults receiving hemodialysis. Res. Nurs. Health.

[66] Wild M.G., Wallston K.A., Green J.A., Beach L.B., Umeukeje E., Nunes J.A.W., Ikizler T.A., Steed J., Cavanaugh K.L. (2017). The Perceived Medical Condition Self-Management Scale can be applied to patients with chronic kidney disease. Kidney Int..

[67] Hair J.F., Howard M.C., Nitzl C. (2020). Assessing measurement model quality in PLS-SEM using confirmatory composite analysis. J. Bus. Res..

[68] Henseler J., Ringle C.M., Sarstedt M. (2014). A new criterion for assessing discriminant validity in variance-based structural equation modeling. J. Acad. Mark. Sci..

[69] Hair J.F., Risher J.J., Sarstedt M., Ringle C.M. (2019). When to use and how to report the results of PLS-SEM. Eur. Bus. Rev..

[70] Hayes A.F. (2018). Introduction to Mediation, Moderation, and Conditional Process Analysis.

[71] St-Jules D.E., Woolf K., Pompeii M.L., Sevick M.A. (2016). Exploring Problems in Following the Hemodialysis Diet and Their Relation to Energy and Nutrient Intakes: The BalanceWise Study. J. Ren. Nutr..

[72] Baron R.M., Kenny D.A. (1986). The moderator–mediator variable distinction in social psychological research: Conceptual, strategic, and statistical considerations. J. Pers. Soc. Psychol..

[73] Green J.A., Mor M.K., Shields A.M., Sevick M.A., Palevsky P.M., Fine M.J., Arnold R.M., Weisbord S.D. (2011). Prevalence and demographic and clinical associations of health literacy in patients on maintenance hemodialysis. Clin. J. Am. Soc. Nephrol..

[74] Cavanaugh K.L., Wingard R.L., Hakim R.M., Eden S., Shintani A., Wallston K.A., Huizinga M.M., Elasy T.A., Rothman R.L., Ikizler T.A. (2010). Low health literacy associates with increased mortality in ESRD. J. Am. Soc. Nephrol..

[75] Taylor D., Bradley J.A., Bradley C., Draper H., Johnson R., Metcalfe W., Oniscu G., Robb M., Tomson C., Watson C. (2016). Limited health literacy in advanced kidney disease. Kidney Int..

[76] Khor B.-H., China K., Halim A.G., Zaher Z.M.M., Ahmad G., Bavanandan S., Visvanathan R., Yahya R., Goh B.L., Bee B.-C. (2018). The state of nutrition care in outpatient hemodialysis settings in Malaysia: A nationwide survey. BMC Health Serv. Res..

[77] Karupaiah T., Morad Z. (2007). Perspectives on the Nutritional Management of Renal Disease in Asia: People, Practice, and Programs. J. Ren. Nutr..

[78] Pafili Z., Maridaki M., Giannaki C.D., Karatzaferi C., Liakopoulos V., Eleftheriadis T., Stefanidis I., Sakkas G.K. (2019). Phosphorus nutritional knowledge among dialysis health care providers and patients: A multicenter observational study. Clin. Nutr. ESPEN.

[79] Stevenson J., Tong A., Campbell K.L., Craig J.C., Lee V.W.S. (2018). Perspectives of healthcare providers on the nutritional management of patients on haemodialysis in Australia: An interview study. BMJ Open.

[80] Tsai W.-C., Yang J.-Y., Luan C.-C., Wang Y.-J., Lai Y.-C., Liu L.-C., Peng Y.-S. (2015). Additional benefit of dietitian involvement in dialysis staffs-led diet education on uncontrolled hyperphosphatemia in hemodialysis patients. Clin. Exp. Nephrol..

[81] Jin X.W., Slomka J., E Blixen C. (2002). Cultural and clinical issues in the care of Asian patients. Clevel. Clin. J. Med..

[82] Easton P.M., Entwistle V., Williams B. (2013). How the stigma of low literacy can impair patient-professional spoken interactions and affect health: Insights from a qualitative investigation. BMC Health Serv. Res..

[83] Lutz E.R., Costello K.L., Jo M., Gilet C.A., Hawley J.M., Bridgman J.C., Song M.-K. (2014). A systematic evaluation of websites offering information on chronic kidney disease. Nephrol. Nurs..

[84] Dageforde L.A., Cavanaugh K.L. (2013). Health literacy: Emerging evidence and applications in kidney disease care. Adv. Chronic Kidney Dis..

[85] Chesser A.K., Woods M.N.K., Smothers K., Rogers N. (2016). Health Literacy and Older Adults. Gerontol. Geriatr. Med..

[86] Cutilli C.C. (2007). Health Literacy in Geriatric Patients. Orthop. Nurs..

[87] Weiss B.D. (2007). Health Literacy and Patient Safety: Help Patients Understand. Manual for Clinicians.

[88] Jain D., Green J.A. (2016). Health literacy in kidney disease: Review of the literature and implications for clinical practice. World J. Nephrol..

[89] Volandes A.E., Paasche-Orlow M.K. (2007). Health Literacy, Health Inequality and a Just Healthcare System. Am. J. Bioeth..

[90] Ostini R., Kairuz T.E. (2013). Investigating the association between health literacy and non-adherence. Int. J. Clin. Pharm..

[91] Lindquist L.A., Go L., Fleisher J., Jain N., Friesema E., Baker D.W. (2011). Relationship of Health Literacy to Intentional and Unintentional Non-Adherence of Hospital Discharge Medications. J. Gen. Intern. Med..

[92] Health literacy: Report of the Council on Scientific Affairs (1999). Ad Hoc Committee on Health Literacy for the Council on Scientific Affairs, American Medical Association. JAMA.

[93] Lastrucci V., Lorini C., Caini S., Bonaccorsi G. (2019). Florence Health Literacy Research Group Health literacy as a mediator of the relationship between socioeconomic status and health: A cross-sectional study in a population-based sample in Florence. PLoS ONE.

[94] Pelikan J., Ganahl K., Roethlin F. (2018). Health literacy as a determinant, mediator and/or moderator of health: Empirical models using the European Health Literacy Survey dataset. Glob. Health Promot..

[95] Paasche-Orlow M.K., Wolf M.S. (2007). The Causal Pathways Linking Health Literacy to Health Outcomes. Am. J. Health Behav..

[96] Boulware L.E., Carson K.A., Troll M.U., Powe N.R., Cooper L.A. (2009). Perceived Susceptibility to Chronic Kidney Disease among High-risk Patients Seen in Primary Care Practices. J. Gen. Intern. Med..

[97] Khawnekar D., Jeanes Y., Gibson E., Held I., Rutherford P. Does Dietary Knowledge in Patients on Haemodialysis Influence Compliance?. Proceedings of the OCE Malnutrition Matters, Joint BAPEN and Nutrition Society.

[98] DeWalt D.A., Callahan L.F., Hawk V.H., Broucksou K.A., Hink A., Rudd R., Brach C., Health Literacy Universal Precautions Toolkit (2010). Prepared by North Carolina Network Consortium. The Cecil G. USA: Sheps Center for Health Services Research. https://www.ahrq.gov/sites/default/files/wysiwyg/professionals/quality-patient-safety/quality-resources/tools/literacy-toolkit/healthliteracytoolkit.pdf.

[99] Usherwoord T. (2017). Encouraging Adherence to Long-Term Medication. Aust. Prescr..

[100] Náfrádi L., Galimberti E., Nakamoto K., Schulz P.J. (2016). Intentional and unintentional medication non-adherence in hypertension: The role of health literacy, empowerment and medication beliefs. J. Public Health Res..

[101] Wroe A.L. (2002). Intentional and Unintentional Nonadherence: A Study of Decision Making. J. Behav. Med..

[102] Bowling A. (2005). Mode of questionnaire administration can have serious effects on data quality. J. Public Health.

[103] Podsakoff P.M., MacKenzie S.B., Lee J.Y., Podsakoff N.P. (2003). Common method biases in behavioral research: A critical review of the literature and recommended remedies. J. Appl. Psychol..

