# Supplementary material for: Understanding How Nutrition Literacy Links to Dietary Adherence in Patients Undergoing Maintenance Hemodialysis: A Theoretical Exploration using Partial Least Squares Structural Equation Modeling
Source: Int J Environ Res Public Health. 2020 Oct 14;17(20):7479. doi: 10.3390/ijerph17207479 (PMC7602379; doi:10.3390/ijerph17207479)
Supplement: Supplementary file 1 [file ijerph-17-07479-s001.zip › IJERPH Supplementary Figure S1.docx]

**Supplementary Figure S1: Flowchart of Patient Recruitment**


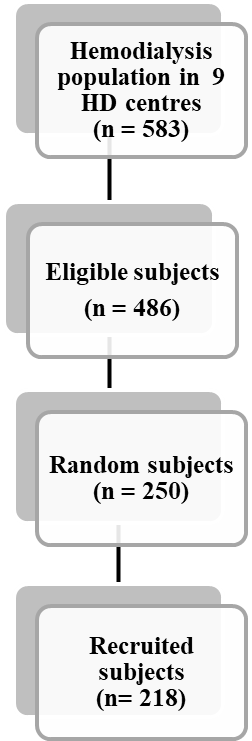


**Refused consent (n=32)**

**Excluded patients (n=97)**

- **Dialyzed < 6 months (n = 37)**
- **Slurred speech (n = 2)**
- **Blind (n = 4)**
- **Frail & severely ill (n = 25)**
- **Admitted (n = 9)**
- **Deceased (n = 8)**
- **Undergone transplant (n = 1)**
- **Changed of modality (n = 2)**
- **Transferred out (n = 2)**
- **Stunted growth (n = 1)**
- **Involved in oral nutrition supplementation studies (n = 6)**
