# Supplementary material for: Understanding How Nutrition Literacy Links to Dietary Adherence in Patients Undergoing Maintenance Hemodialysis: A Theoretical Exploration using Partial Least Squares Structural Equation Modeling
Source: Int J Environ Res Public Health. 2020 Oct 14;17(20):7479. doi: 10.3390/ijerph17207479 (PMC7602379; doi:10.3390/ijerph17207479)
Supplement: Supplementary file 1 [file ijerph-17-07479-s001.zip › IJERPH Supplementary Figure S2.docx]

**Supplementary Figure S2: Causal Model Linking Nutrition Literacy to Dietary Adherence in HD Patients**

**Nutrition Literacy**

**Dietary Adherence**

**Self-efficacy**

**Self-management skills**

**Risk Factors**

- Age
- Education level
- Dialysis vintage

**Mediators**
