# Supplementary material for: Understanding How Nutrition Literacy Links to Dietary Adherence in Patients Undergoing Maintenance Hemodialysis: A Theoretical Exploration using Partial Least Squares Structural Equation Modeling
Source: Int J Environ Res Public Health. 2020 Oct 14;17(20):7479. doi: 10.3390/ijerph17207479 (PMC7602379; doi:10.3390/ijerph17207479)
Supplement: Supplementary file 1 [file ijerph-17-07479-s001.zip › IJERPH Supplementary Table S1.docx]

**Supplementary Table S1**

This scale contains 8 questions to assess your dialysis-specific nutrition literacy skills (poor/fair/good). Please answer all the questions below completely and honestly.

| **Dialysis Specific Nutrition Literacy Scale (DSNLS)** | **Poor** | **Fair** | **Good** |
| --- | --- | --- | --- |
| 1. How would you rate your ability to obtain information on diet-related health complications from health-care personnel?   Please specify:   \| 1. Medical doctor \| 1. Nurse \| 1. Medical assistant \| 1. Dietitian \| \| --- \| --- \| --- \| --- \| \| 1. Others: _____________ \| \|  \|  \| | 1 | 2 | 3 |
| 1. How would you rate your understanding on diet-related health complications in dialysis on printed education materials?  \| 1. Diet low in energy & protein \| 1. Diet high in sodium \| \| --- \| --- \| \| 1. Diet high in potassium \| 1. Diet high in phosphorus \|   **Please rate “3” if you know at least three complications; rate “2” if you know at least two complications; otherwise please rate “1”.* | 1 | 2 | 3 |
| 1. How would you rate your ability to evaluate your diet-related health risk with reference to your laboratory results? | 1 | 2 | 3 |
| 1. How would you rate your ability to obtain tips on dialysis diet from health-care professionals?   Please specify:   \| 1. Medical doctor \| 1. Nurse \| 1. Medical assistant \| 1. Dietitian \| \| --- \| --- \| --- \| --- \| \| 1. Others: _____________ \| \|  \|  \| | 1 | 2 | 3 |
| 1. How would you rate your ability to understand the concept of dialysis diet on printed education materials?  \| 1. Types of nutrient that need to be consumed sufficiently. \| \| --- \| \| 1. Types of nutrient that need to be limited. \| \| 1. Dietary modification to reduce minerals content of foods. \|   **Please rate “3” if you know all the three concepts; rate “2” if you know at least two concepts; otherwise please rate “1”.* | 1 | 2 | 3 |
| 1. How would you rate your ability to judge the nutrient levels (high sodium, potassium & phosphorus content) in food items? | 1 | 2 | 3 |
| 1. How would you rate your ability to obtain information on dialysis diet from sources other than healthcare personnel?   Please specify:   \| a) Internet \| b) Family members \| b) Peers/Friends; \| \| --- \| --- \| --- \| \| c) Others: ______________ \| \|  \| | 1 | 2 | 3 |
| 1. How would you rate your ability to judge if the information on dialysis diet from non-medical sources (e.g. internet, friends and etc.) is reliable? | 1 | 2 | 3 |
