# Supplementary material for: Understanding How Nutrition Literacy Links to Dietary Adherence in Patients Undergoing Maintenance Hemodialysis: A Theoretical Exploration using Partial Least Squares Structural Equation Modeling
Source: Int J Environ Res Public Health. 2020 Oct 14;17(20):7479. doi: 10.3390/ijerph17207479 (PMC7602379; doi:10.3390/ijerph17207479)
Supplement: Supplementary file 1 [file ijerph-17-07479-s001.zip › IJERPH Supplementary Table S2.docx]

**Supplementary Table S2**

**Sample questionnaire of dialysis-related dietary knowledge questionnaire (DDKQ)**

| 1. Excess intake of potassium _____________   (1) is harmful to bone (3) raise blood pressure  (2) is harmful to heart (4) not sure |
| --- |
| 1. Excess intake of sodium _____________   (1) is harmful to bone (3) raise blood pressure  (2) increase body weight (4) not sure |
| 1. Excess intake of phosphate _____________   (1) is harmful to bone (3) causes breathing difficulty  (2) raise blood pressure (4) not sure |
| 1. Which of the following food item(s)/beverage(s) is/are high in potassium?  \| Items \| Yes \| No \| Not sure \| \| --- \| --- \| --- \| --- \| \| 1. Chocolate beverages \| 1 \| 2 \| 0 \| \| 1. Syrup drinks \| 1 \| 2 \| 0 \| \| 1. Coconut water \| 1 \| 2 \| 0 \| \| 1. Bean sprout \| 1 \| 2 \| 0 \| \| 1. Sawi \| 1 \| 2 \| 0 \| \| 1. Petai \| 1 \| 2 \| 0 \| \| 1. Potato \| 1 \| 2 \| 0 \| \| 1. Banana \| 1 \| 2 \| 0 \| \| 1. Grapes \| 1 \| 2 \| 0 \| \| 1. Apple \| 1 \| 2 \| 0 \| \| 1. Durian \| 1 \| 2 \| 0 \| |
| 1. Which of the following food item(s)/beverage(s) is/are high in phosphate?  \| Food Items \| Yes \| No \| Not sure \| \| --- \| --- \| --- \| --- \| \| 1. Teh Tarik \| 1 \| 2 \| 0 \| \| 1. Milk \| 1 \| 2 \| 0 \| \| 1. Orange juice \| 1 \| 2 \| 0 \| \| 1. Instant noodle \| 1 \| 2 \| 0 \| \| 1. Roti Canai \| 1 \| 2 \| 0 \| \| 1. Kuey Teow \| 1 \| 2 \| 0 \| \| 1. Egg white \| 1 \| 2 \| 0 \| \| 1. Fish \| 1 \| 2 \| 0 \| |
| 1. Which of the following food(s) is/are high in sodium?  \| Food Items \| Yes \| No \| Not sure \| \| --- \| --- \| --- \| --- \| \| 1. Anchovy \| 1 \| 2 \| 0 \| \| 1. Sausage \| 1 \| 2 \| 0 \| \| 1. Fast food \| 1 \| 2 \| 0 \| |
