# Supplementary material for: Understanding How Nutrition Literacy Links to Dietary Adherence in Patients Undergoing Maintenance Hemodialysis: A Theoretical Exploration using Partial Least Squares Structural Equation Modeling
Source: Int J Environ Res Public Health. 2020 Oct 14;17(20):7479. doi: 10.3390/ijerph17207479 (PMC7602379; doi:10.3390/ijerph17207479)
Supplement: Supplementary file 1 [file ijerph-17-07479-s001.zip › IJERPH Supplementary Table S3.docx]

**Supplementary Table S3**

**Sample of self-developed Dialysis Diet-related Health Belief Questionnaire (DDHBQ) to assess the patient’s perceptions of dietary adherence.**

**Perceived Benefits of Dietary Adherence**

| **Items** | **Strongly disagree** | **Slightly disagree** | **Neutral** | **Agree** | **Strongly agree** |
| --- | --- | --- | --- | --- | --- |
| 1. Adhering to renal diet can help to prevent bone disease. | 1 | 2 | 3 | 4 | 5 |
| 1. Adhering to renal diet can help to prevent heart disease. | 1 | 2 | 3 | 4 | 5 |
| 1. Adhering to renal diet can reduce the risk of hospitalization. | 1 | 2 | 3 | 4 | 5 |
| 1. Adhering to renal diet can help to prevent malnutrition. | 1 | 2 | 3 | 4 | 5 |
| 1. When I follow renal diet, I feel better. | 1 | 2 | 3 | 4 | 5 |
| 1. Eating a renal diet helps keep my blood pressure down. | 1 | 2 | 3 | 4 | 5 |
| 1. Adhering to renal diet reduces the feeling of thirst. | 1 | 2 | 3 | 4 | 5 |

**Perceived Barriers of Dietary Adherence**

| **Items** | **Strongly disagree** | **Slightly disagree** | **Neutral** | **Agree** | **Strongly agree** |
| --- | --- | --- | --- | --- | --- |
| 1. I cannot adhere to renal diet due to the lack of dietary knowledge. | 1 | 2 | 3 | 4 | 5 |
| 1. Adhere to renal diet is difficult as it affects my food preference. | 1 | 2 | 3 | 4 | 5 |
| 1. It is difficult to understand how to follow a renal diet. | 1 | 2 | 3 | 4 | 5 |
| 1. The taste of renal diet is not good. | 1 | 2 | 3 | 4 | 5 |
| 1. Following a renal diet is hard to do when I go out to eat | 1 | 2 | 3 | 4 | 5 |

**Perceived Seriousness of Health Complications attributed to Dietary Non-adherence**

| **Items** | **Strongly disagree** | **Slightly disagree** | **Neutral** | **Agree** | **Strongly agree** |
| --- | --- | --- | --- | --- | --- |
| 1. I am worried to be admitted to hospital. | 1 | 2 | 3 | 4 | 5 |
| 1. The thought of getting heart disease scares me. | 1 | 2 | 3 | 4 | 5 |
| 1. Getting high blood pressure is terrifying. | 1 | 2 | 3 | 4 | 5 |
| 1. I am afraid to even think about getting bone disease. | 1 | 2 | 3 | 4 | 5 |
| 1. Being malnourished is bad. | 1 | 2 | 3 | 4 | 5 |

**Perceived Susceptibility of Health Complications attributed to Dietary Non-adherence**

| **Items** | **Strongly disagree** | **Slightly disagree** | **Neutral** | **Agree** | **Strongly agree** |
| --- | --- | --- | --- | --- | --- |
| 1. I am likely to be hospitalized in the near future. | 1 | 2 | 3 | 4 | 5 |
| 1. It is likely that my blood pressure will get worse in the future | 1 | 2 | 3 | 4 | 5 |
| 1. I may experience malnutrition in the future. | 1 | 2 | 3 | 4 | 5 |
| 1. The likelihood for me to get heart disease in the near future is high. | 1 | 2 | 3 | 4 | 5 |
| 1. The likelihood for me to get bone disease in the near future is high. | 1 | 2 | 3 | 4 | 5 |

**Perceived Self-efficacy of Dietary Adherence**

| **Items** | **Not at all confident** | **Little confident** | **Neither** | **Somewhat confident** | **Very confident** |
| --- | --- | --- | --- | --- | --- |
| 1. Staying on a renal diet when I am busy or in a rush. | 1 | 2 | 3 | 4 | 5 |
| 1. Staying on a renal diet when no one at home is on it. | 1 | 2 | 3 | 4 | 5 |
| 1. Staying on a renal diet when I am eating outside. | 1 | 2 | 3 | 4 | 5 |
| 1. Staying on a renal diet on special occasions or holidays. | 1 | 2 | 3 | 4 | 5 |
| 1. Knowing what kind of foods I should eat on a renal diet. | 1 | 2 | 3 | 4 | 5 |
| 1. Knowing what suitable foods to buy at the grocery stores in regard to my health condition. | 1 | 2 | 3 | 4 | 5 |
