# Supplementary material for: Understanding How Nutrition Literacy Links to Dietary Adherence in Patients Undergoing Maintenance Hemodialysis: A Theoretical Exploration using Partial Least Squares Structural Equation Modeling
Source: Int J Environ Res Public Health. 2020 Oct 14;17(20):7479. doi: 10.3390/ijerph17207479 (PMC7602379; doi:10.3390/ijerph17207479)
Supplement: Supplementary file 1 [file ijerph-17-07479-s001.zip › IJERPH Supplementary Table S4.docx]

**Supplementary Table S1. Results of Confirmatory Composite Analysis**

| **Constructs & Items** | **Standardized Factor loading (> 0.5)** | **(AVE ≥ 0.5)** | **(CR ≥ 0.7)** | **Discriminant Validity (HTMT ratio < 0.85) ^a^** |  |
| --- | --- | --- | --- | --- | --- |
| **Nutrition Literacy** |  | 0.615 | 0.927 | 0.105-0.655 | |
| - DSNLS 1 | 0.789 |  |  |  | |
| - DSNLS 2 | 0.815 |  |  |  | |
| - DSNLS 3 | 0.801 |  |  |  | |
| - DSNLS 4 | 0.752 |  |  |  | |
| - DSNLS 5 | 0.838 |  |  |  | |
| - DSNLS 6 | 0.820 |  |  |  | |
| - DSNLS 7 | 0.679 |  |  |  | |
| - DSNLS 8 | 0.769 |  |  |  | |
| **Perceived Benefit** |  | 0.540 | 0.854 | 0.144-0.564 | |
| - BE 1 | 0.717 |  |  |  | |
| - BE 2 | 0.834 |  |  |  | |
| - BE 3 | 0.753 |  |  |  | |
| - BE 5 | 0.686 |  |  |  | |
| - BE 6 | 0.674 |  |  |  | |
| **Perceived Seriousness** |  | 0.575 | 0.844 | 0.062-0.564 | |
| - SE 1 | 0.668 |  |  |  | |
| - SE 2 | 0.791 |  |  |  | |
| - SE 3 | 0.814 |  |  |  | |
| - SE 4 | 0.754 |  |  |  | |
| **Perceived Susceptibility** |  | 0.537 | 0.816 | 0.102-0.279 | |
| - SU 1 | 0.623 |  |  |  | |
| - SU 3 | 0.578 |  |  |  | |
| - SU 4 | 0.699 |  |  |  | |
| - SU 5 | 0.967 |  |  |  | |
| **Perceived Self-Efficacy** |  | 0.526 | 0.868 | 0.142-0.569 | |
| - EF 1 | 0.759 |  |  |  | |
| - EF 2 | 0.779 |  |  |  | |
| - EF 3 | 0.648 |  |  |  | |
| - EF 4 | 0.588 |  |  |  | |
| - EF 5 | 0.790 |  |  |  | |
| - EF 6 | 0.763 |  |  |  | |
| **Self-Management Skills** |  | 0.562 | 0.909 | 0.102-0.655 | |
| - PKDSMS 1 | 0.673 |  |  |  | |
| - PKDSMS 2 | 0.826 |  |  |  | |
| - PKDSMS 3 | 0.594 |  |  |  | |
| - PKDSMS 4 | 0.507 |  |  |  | |
| - PKDSMS 5 | 0.871 |  |  |  | |
| - PKDSMS 6 | 0.792 |  |  |  | |
| - PKDSMS 7 | 0.823 |  |  |  | |
| - PKDSMS 8 | 0.830 |  |  |  | |
| **Knowledge Barrier** |  | 0.861 | 0.925 | 0.091-0.593 | |
| - BA 1 | 0.930 |  |  |  | |
| - BA 3 | 0.925 |  |  |  | |
| **Food Preference** |  | 0.686 | 0.813 | 0.091-0.571 | |
| - BA 2 | 0.812 |  |  |  | |
| - BA 4 | 0.843 |  |  |  | |

Note: Only valid items were presented; Single item is exempted from assessment; AVE: Average Variance Extracted; CR: Composite Reliability; HTMT ratio: Heterotrait-Monotrait ratio of correlation; ^a^ Data presented as range (min-max)
